# A Breast Imaging Case of Steatocystoma Multiplex: A Rare Condition Involving Multiple Anatomic Regions

**DOI:** 10.7759/cureus.27756

**Published:** 2022-08-07

**Authors:** Victoria Reick-Mitrisin, Abhinaya Reddy, Biren A Shah

**Affiliations:** 1 Medical Education, A.T. Still University-Kirksville College of Osteopathic Medicine (ATSU-KCOM), Kirksville, USA; 2 Diagnostic Radiology, Detroit Medical Center/Wayne State University, Detroit, USA

**Keywords:** ultrasound scan, colored flow doppler ultrasound, subcutaneous cyst, recurrent cyst, breast screening, clinical dermatology, general radiology

## Abstract

Steatocystoma multiplex is an uncommon disease consisting of multiple cysts erupting over the chest, arms, axilla, and neck. It is of unknown incidence and can occur as a spontaneous mutation or inherited in an autosomal dominant pattern.

A 47-year-old female with a past medical history only significant for multiple skin infections presented for a routine mammogram. Her imaging showed multiple circumscribed oil cysts. When contacted regarding the results, the patient said she has noticed multiple cysts presenting on her inner arms, chest, and trunk, of which one would occasionally exsanguinate oily material. She denies any other associated symptoms and says that she is the only member of her family to have these symptoms. The patient was informed of her diagnosis and requested to follow up with her primary care physician to monitor her symptoms.

In summary, steatocystoma multiplex is a rare benign condition that can present similarly to a variety of other pathologies. It is important to note the unique clinical features of steatocystoma multiplex in order to prevent unnecessary and costly workup for patients who have this benign condition.

## Introduction

Steatocystoma multiplex is an uncommon disease consisting of multiple cysts erupting over the chest, arms, axilla, and neck, most often inherited in an autosomal dominant fashion [1]. These lesions are largely asymptomatic but may erupt exuding an oily substance if the cyst is close to the skin surface [2]. Many of the features of this condition overlap with a variety of diseases such as eruptive vellus hair cyst (EVHC) and acne vulgaris. It is therefore important to differentiate, as the workup and treatment differ widely [2,3]. This case report outlines a presentation of steatocystoma multiplex in order to draw attention to the unique clinical features of this rare condition.

## Case presentation

A 47-year-old female with a past medical history only significant for recurrent skin infections presented for a routine mammogram with no symptoms or concerns.

**Figure 1:**
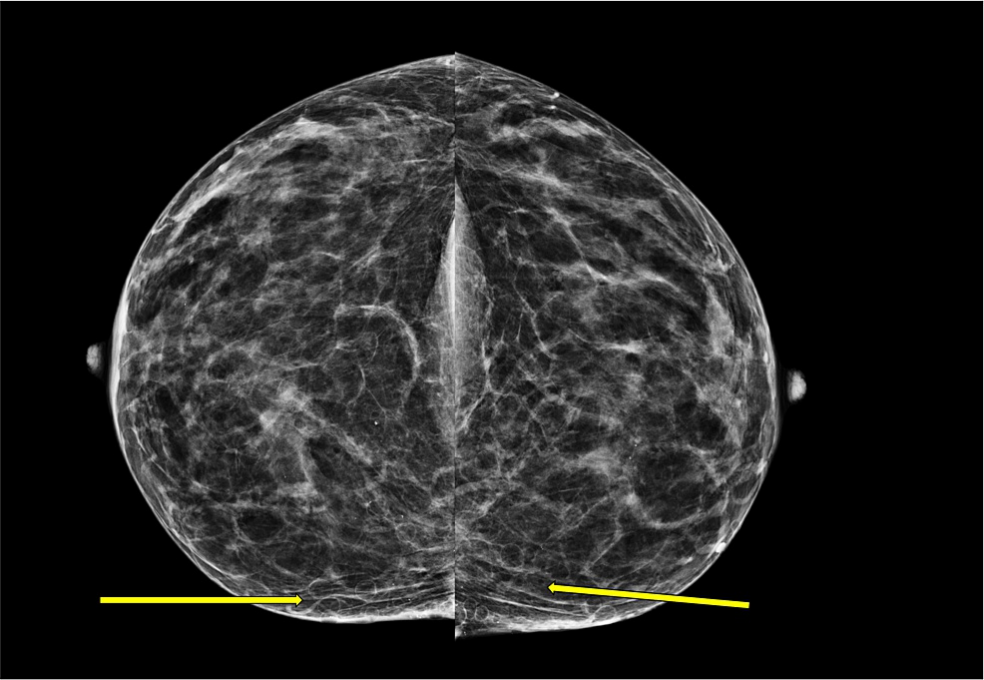
This craniocaudal mammographic image shows multiple benign oil cysts within the patient’s breasts bilaterally consistent with a diagnosis of steatocystoma multiplex (arrows).

**Figure 2:**
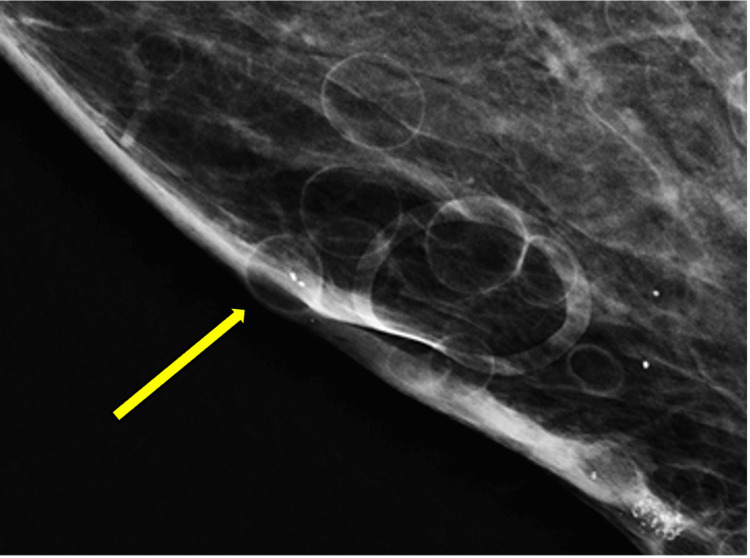
Zoomed-in craniocaudal magnified view of the patient’s right breast on mammography. Multiple subcutaneous cysts are visualized (arrow).

**Figure 3:**
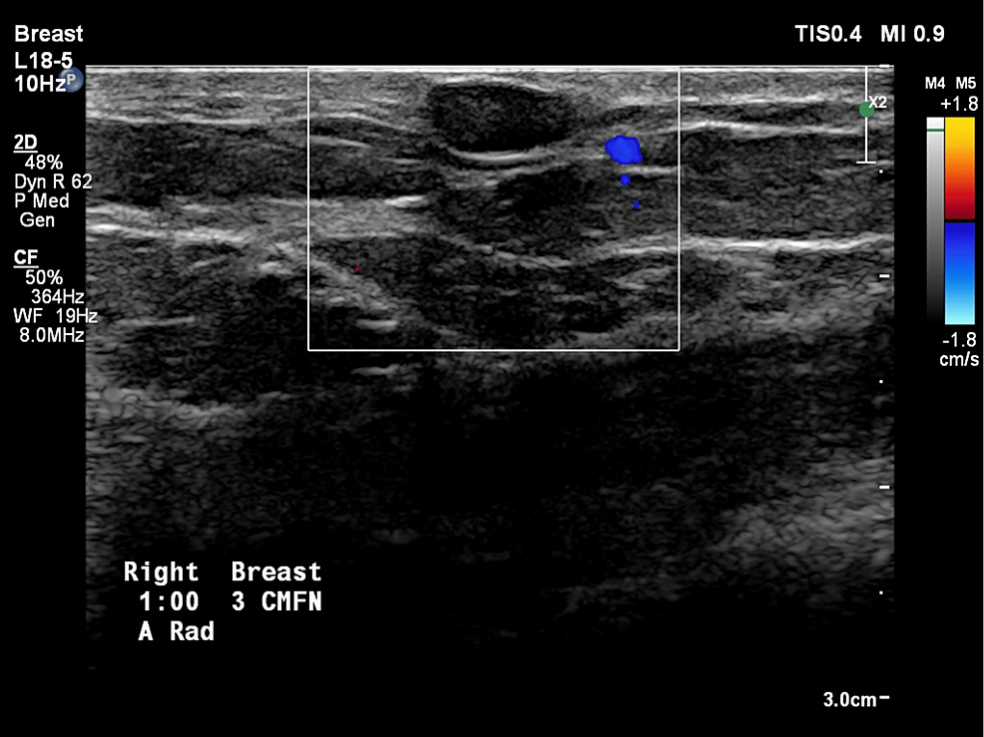
Doppler ultrasound image of oil cysts in the patient’s right breast depicting a circumscribed cyst in the 1 o’clock position, 3 cm from the nipple.

The patient’s screening mammogram was performed (Figures 1, 2), and innumerable oil cysts are visualized in both breasts, predominantly in the medial aspect. The patient had a diagnostic mammogram with targeted bilateral breast ultrasound, which showed multiple oval anechoic cystic masses consistent with mammographic findings of multiple oil cysts. A follow-up ultrasound was done (Figure 3), which showed a circumscribed, avascular oil cyst.

**Figure 4:**
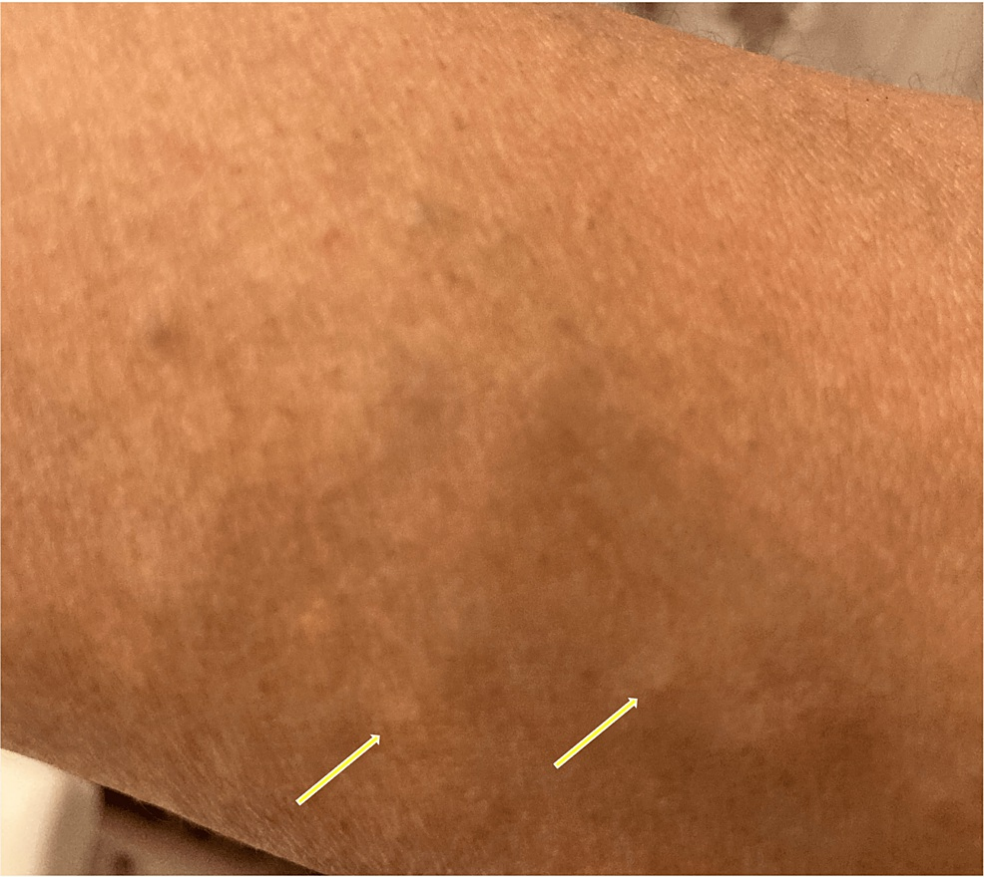
Multiple small subcutaneous cysts are present on the patient’s inner forearm (arrows). The patient’s symptoms extend to her chest and trunk as well (not depicted).

On inspection of the patient’s chart, she did not have a history of breast trauma, previous breast procedures, or breast complaints. The patient was contacted after her imaging results. She denied any significant medical history or any family history of breast or skin pathology at this time. She did note that since early adulthood, she persistently had multiple “bumps” on her trunk, chest, and inner arms, representing subcutaneous cysts that slowly increased in number over time (Figure 4). She also notes one subcutaneous cyst on her chest that will occasionally erupt and exsanguinate oily fluid. She denied any other associated symptoms or concerns with regard to her cysts and says that she is the only one in her family with these symptoms.

The patient’s primary care provider was contacted regarding the findings and will continue to monitor her symptoms on an outpatient basis.

## Discussion

Steatocystoma multiplex is an uncommon benign condition that has overlapping clinical features with a variety of other pathologies. Cysts commonly occur subcutaneously on the arms, axilla, and trunk starting at puberty. It is important to be aware of the unique features of this condition in order to differentiate it from other possibly more serious diseases. By correctly identifying this diagnosis, patients can also be spared undue psychological distress, unnecessary workup, and hospital fees. This condition has overlapping features with diseases such as eruptive vellus hair cysts, trichofolliculoma, and syringoma [4,5]. It can also appear similar to lipomas, fat necrosis, galactoceles, and epidermal inclusion cysts, in addition to many other dermatologic conditions. This is why if the case is not clinically clear, further histological evidence may be warranted [6].

This condition involves small, non-painful subcutaneous cysts appearing most commonly on the trunk, upper extremities, and axilla starting in puberty [1,7]. The histological features of steatocystoma multiplex consist of stratified squamous epithelial walls with an irregularly lined eosinophilic center [1]. While many cases are found to be sporadic mutations, there is a strong association with the KRT17 gene, which is of an autosomal dominant inheritance pattern. Other mutations have also been associated with this condition, including N92S, R94H, and R94C [8]. It does not appear to have an increased prevalence in a specific gender or ethnic group [9]. Imaging findings in steatocystoma multiplex include mammography showing multiple oil cysts (small, round, circumscribed cysts with a central fat density). Ultrasound imaging shows multiple anechoic cysts with posterior acoustic enhancement.

If the patient’s clinical presentation is suspicious for multiple conditions, steatocystoma multiplex can be differentiated using histological features. One study analyzed the histological features of 67 cases with steatocystoma multiplex and found that all cases had an eosinophilic cuticle with no glandular layer, with a minority of cases also containing hair follicles, keratin, and smooth muscle [2]. The walls of the cysts are stratified squamous epithelium with an uneven eosinophilic border at the center [1].

Once the diagnosis of steatocystoma multiplex is confirmed, patients do not require further imaging or workup, as the condition is benign and does not require intervention. However, patients often experience psychological distress from their symptoms [10]. If the patient wishes to pursue further treatment for cysts, there are a variety of treatment modalities, with no specific gold standard or preferred option. Treatment should rather be geared toward the patient’s individual presentation [3]. Options include cryotherapy, aspiration, laser therapy, or surgical resection. Treatment is also aimed at preventing secondary skin infections.

## Conclusions

Steatocystoma multiplex is a rare condition with an unknown prevalence in the population. It has no gender or ethnic predilection and is due to a sporadic or autosomal dominant mutation with many different associated genes.

While this condition is benign, it is important to have a clinical suspicion for this condition, as it has overlapping clinical features with a wide array of other dermatologic conditions that require different treatment modalities. Additionally, it is important to note as this condition is benign and requires no further workup. A variety of treatment options aimed at cosmetic changes and preventing infection are available if the patient is experiencing psychological distress due to symptoms.
